# Synthesis and Preclinical Evaluation of ^177^Lu-Labeled Radiohybrid PSMA Ligands for Endoradiotherapy of Prostate Cancer

**DOI:** 10.2967/jnumed.121.263371

**Published:** 2022-10

**Authors:** Alexander Wurzer, Jan-Philip Kunert, Sebastian Fischer, Veronika Felber, Roswitha Beck, Francesco de Rose, Calogero D’Alessandria, Wolfgang Weber, Hans-Jürgen Wester

**Affiliations:** 1 Chair of Pharmaceutical Radiochemistry, Technical University of Munich, Garching, Germany; and; 2 Department of Nuclear Medicine, Klinikum Rechts der Isar, Technical University of Munich, Munich, Germany

**Keywords:** radiohybrid, rhPSMA, PSMA, radioligand therapy, prostate cancer

## Abstract

The prostate-specific membrane antigen (PSMA)–targeted radiohybrid (rh) ligand [^177^Lu]Lu-rhPSMA-7.3 has recently been assessed in a pretherapeutic dosimetry study on prostate cancer patients. In comparison to [^177^Lu]Lu-PSMA I&T, application of [^177^Lu]Lu-rhPSMA-7.3 resulted in a significantly improved tumor dose but also higher kidney accumulation. Although rhPSMA-7.3 has been initially selected as the lead compound for diagnostic application based on the characterization of its gallium complex, a systematic comparison of the most promising ^177^Lu-labeled rhPSMA ligands is still missing. Thus, this study aimed to identify the rhPSMA ligand with the most favorable pharmacokinetics for ^177^Lu-radioligand therapy. **Methods:** The 4 isomers of [^177^Lu]Lu-rhPSMA-7 (namely [^177^Lu]Lu-rhPSMA-7.1, -7.2, -7.3, and -7.4), along with the novel radiohybrid ligands [^177^Lu]Lu-rhPSMA-10.1 and -10.2, were compared with the state-of-the-art compounds [^177^Lu]Lu-PSMA I&T and [^177^Lu]Lu-PSMA-617. The comparative evaluation comprised affinity studies (half-maximal inhibitory concentration) and internalization experiments on LNCaP cells, as well as lipophilicity measurements. In addition, we determined the apparent molecular weight (AMW) of each tracer as a parameter for human serum albumin (HSA) binding. Biodistribution studies and small-animal SPECT imaging were performed on LNCaP-tumor bearing mice at 24 h after injection. **Results:** ^177^Lu labeling of the radiohybrids was performed according to the established procedures for the currently established PSMA-targeted ligands. All ligands showed potent binding to PSMA-expressing LNCaP cells, with affinities in the low nanomolar range and high internalization rates. Surprisingly, the most pronounced differences regarded the HSA-related AMW. Although [^177^Lu]Lu-rhPSMA-7 isomers demonstrated the highest AMW and thus strongest HSA interactions, [^177^Lu]Lu-rhPSMA-10.1 showed an AMW lower than for [^177^Lu]Lu-rhPSMA-7.3 but higher than for the ^177^Lu-labeled references PSMA I&T and PSMA-617. In biodistribution studies, [^177^Lu]Lu-rhPSMA-10.1 exhibited the lowest kidney uptake and fastest excretion from the blood pool of all rhPSMA ligands while preserving a high tumor accumulation. **Conclusion:** Clinical investigation of [^177^Lu]Lu-rhPSMA-10.1 is highly warranted to determine whether the favorable pharmacokinetics observed in mice will also result in high tumor uptake and decreased absorbed dose to kidneys and other nontarget tissues in patients.

## Visual Abstract

**Figure:**
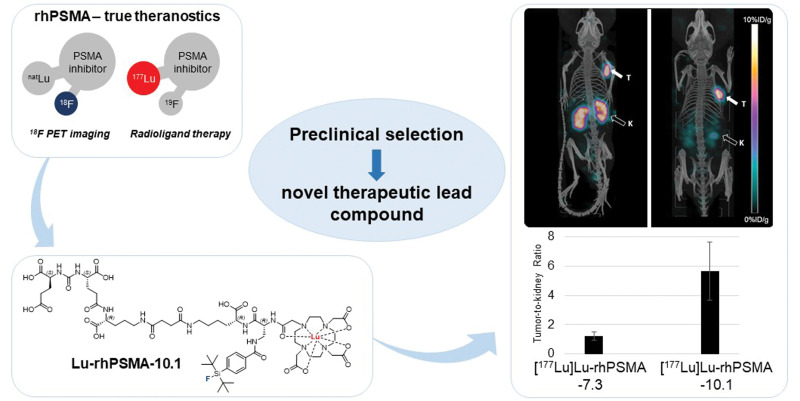

Radioligand therapy (RLT) of metastatic castration-resistant prostate cancer with ligands targeting prostate-specific membrane antigen (PSMA) holds great promise for patients who have exhausted conventional treatment regimens. Currently, ^177^Lu-labeled PSMA-617 (*1*) and PSMA I&T (*2*) are the most extensively evaluated agents in this class and have demonstrated favorable safety and good treatment response rates (*3**,**4*). Although regulatory approval is still awaited for both agents, their application in compassionate-use programs has recently been reaffirmed by the European Association of Nuclear Medicine procedure guidelines for ^177^Lu-PSMA therapy (*5*). [^177^Lu]Lu-PSMA-617 has been evaluated by Novartis in a phase 3 clinical trial (NCT 03511664) on patients with metastatic castration-resistant prostate cancer. In that trial, [^177^Lu]Lu-PSMA-617 was compared with the best standard of care. The investigators recently announced that both primary endpoints—overall and radiographic progression-free survival—were met (*6*). In addition, an ongoing phase 3 trial investigating [^177^Lu]Lu-PSMA I&T (NCT 04647526) is evaluating its efficacy versus abiraterone or enzalutamide in delaying radiographic progression in patients with metastatic castration-resistant prostate cancer after second-line hormonal treatment. Retrospective clinical comparison of ^177^Lu-labeled PSMA-617 and PSMA I&T point toward nearly identical pharmacokinetics for both tracers, and clinical efficacy is assumed to be similar, with no clear advantage to either compound (*7*).

Recently, the novel class of radiohybrid (rh) PSMA-targeted ligands was introduced by our group (*8*). These compounds combine a silicon-fluoride acceptor for ^19^F/^18^F-isotopic exchange radiolabeling and a chelator for complexation of a metal or radiometal (e.g., ^177^Lu, ^68^Ga, or ^225^Ac). Respective ligand pairs of ^18^F/nonradioactive metal and ^19^F/radiometal, such as [^18^F]Lu-rhPSMA and [^177^Lu]Lu-rhPSMA, are chemically identical and thus display identical pharmacokinetics, offering unique possibilities for theranostic applications (Fig. 1).

**FIGURE 1.**
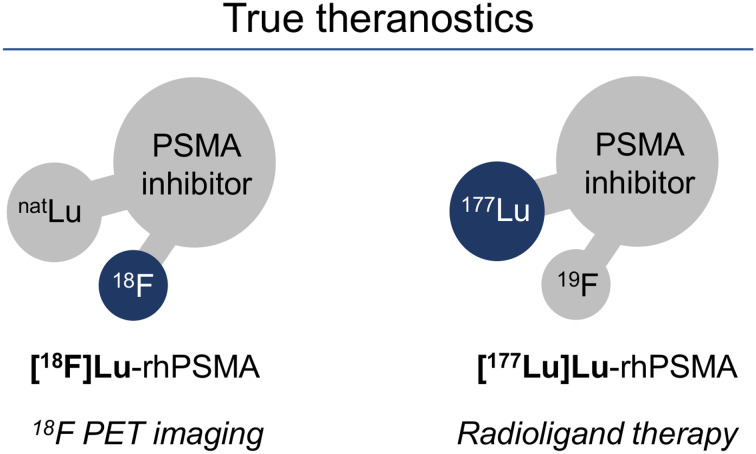
Theranostic radiohybrid concept applied to PSMA-targeted ligands. Molecules offer 2 labeling sites for radionuclides, silicon-fluorine acceptor site for ^18^F labeling via isotopic exchange and chelator for radiometallation. ^18^F-labeled cold lutetium–complexed ligand ([^18^F]Lu-rhPSMA) is chemically identical to ^177^Lu-labeled cold fluorine compound ([^177^Lu]Lu-rhPSMA), therefore representing true theranostic agents for PET imaging and RLT.

For prostate cancer diagnosis, the first clinical evaluations were conducted with the ^18^F-labeled ^nat^Ga chelate of rhPSMA-7, which demonstrated a favorable biodistribution and indicated a high diagnostic performance for N-staging and localization of biochemical recurrence in patients with prostate cancer (*9**–**11*). Since rhPSMA-7 was found to comprise 4 diastereoisomers (rhPSMA-7.1, -7.2, -7.3, and -7.4), a preclinical selection process was initiated that identified [^18^F]Ga-rhPSMA-7.3 (often abbreviated as ^18^F-rhPSMA-7.3) as the novel diagnostic lead compound (*12*) for current phase 3 clinical trials (NCT04186819 and NCT04186845).

For initial evaluation of the radiohybrid technology for therapeutic applications, rhPSMA-7.3 was labeled with ^177^Lu and compared with [^177^Lu]Lu-PSMA I&T in biodistribution and dosimetry studies on mice. Both ligands showed similar uptake in healthy organs, resulting in a similar dose to all major organs, including bone marrow and kidney (*13*). Compared with [^177^Lu]Lu-PSMA I&T, [^177^Lu]Lu-rhPSMA-7.3 exhibited a 2.8- and 4.7-fold higher tumor uptake at 1 and 168 h after injection, respectively, resulting in a significantly higher dose at the tumor and a superior treatment response (*13*).

In a pretherapeutic comparative dosimetry study of ^177^Lu-labeled rhPSMA-7.3 and PSMA I&T on a small patient cohort (*n* = 6, intraindividual comparison), an approximately 2.4-fold higher mean absorbed dose for tumor lesions of the radiohybrid ligand was found, consistent with the preclinical observations (*14*). However, contradictory to animal studies, the mean absorbed dose to different healthy organs was also higher—for example, 2.3-fold higher doses to kidneys and 2.2-fold higher doses to bone marrow for [^177^Lu]Lu-rhPSMA-7.3 versus [^177^Lu]Lu-PSMA I&T. The authors concluded that the radiohybrid tracer holds promise for therapeutic effects similar to those obtained with [^177^Lu]Lu-PSMA I&T while offering potential economic advantages by an approximately 2-fold reduction in the injected radioactive doses (*14*).

The selection of rhPSMA-7.3 as lead compound for diagnostic application was based on the evaluation of gallium chelates [^18^F]Ga-rhPSMA-7.1, -7.2, -7.3, and -7.4 (*12*). Since it is known from the literature that the complex structure of the metal chelate (e.g., [Ga]DOTAGA and [Lu]DOTAGA) within a radioligand can influence its pharmacokinetic properties (*15**,**16*), all ^177^Lu-labeled isomers of rhPSMA-7 have been included in this comparison. The isomers differ in the stereoconfiguration of the diaminopropionic acid branching unit (d-Dap or l-Dap) and the glutamic acid arm at the DOTAGA chelator (*R*- or *S-*DOTAGA: rhPSMA-7.1 [d-Dap–*R*-DOTAGA], rhPSMA-7.2 [l-Dap–*R-*DOTAGA], rhPSMA-7.3 [d-Dap–*S*-DOTAGA], rhPSMA-7.4 [l-Dap–*S*-DOTAGA]).

Given the promising initial data from ^177^Lu-labeled rhPSMA-7.3, the aim of the present study was to evaluate whether other isomers of ^177^Lu-labeled rhPSMA-7 or the closely related compounds [^177^Lu]Lu-rhPSMA-10.1 (d-Dap) and -10.2 (l-Dap) (where DOTA replaces the DOTAGA chelator) might have further improved biodistribution kinetics in normal organs while maintaining the high tumor uptake found with [^177^Lu]Lu-rhPSMA-7.3. We evaluated the ligands in comparison with reference ligands [^177^Lu]Lu-PSMA-617 and [^177^Lu]Lu-PSMA I&T (Fig. 2) in vitro (lipophilicity, half-maximal inhibitory concentration, internalization into LNCaP cells, binding to human serum albumin [HSA]) and in biodistribution studies on LNCaP tumor–bearing mice.

**FIGURE 2.**
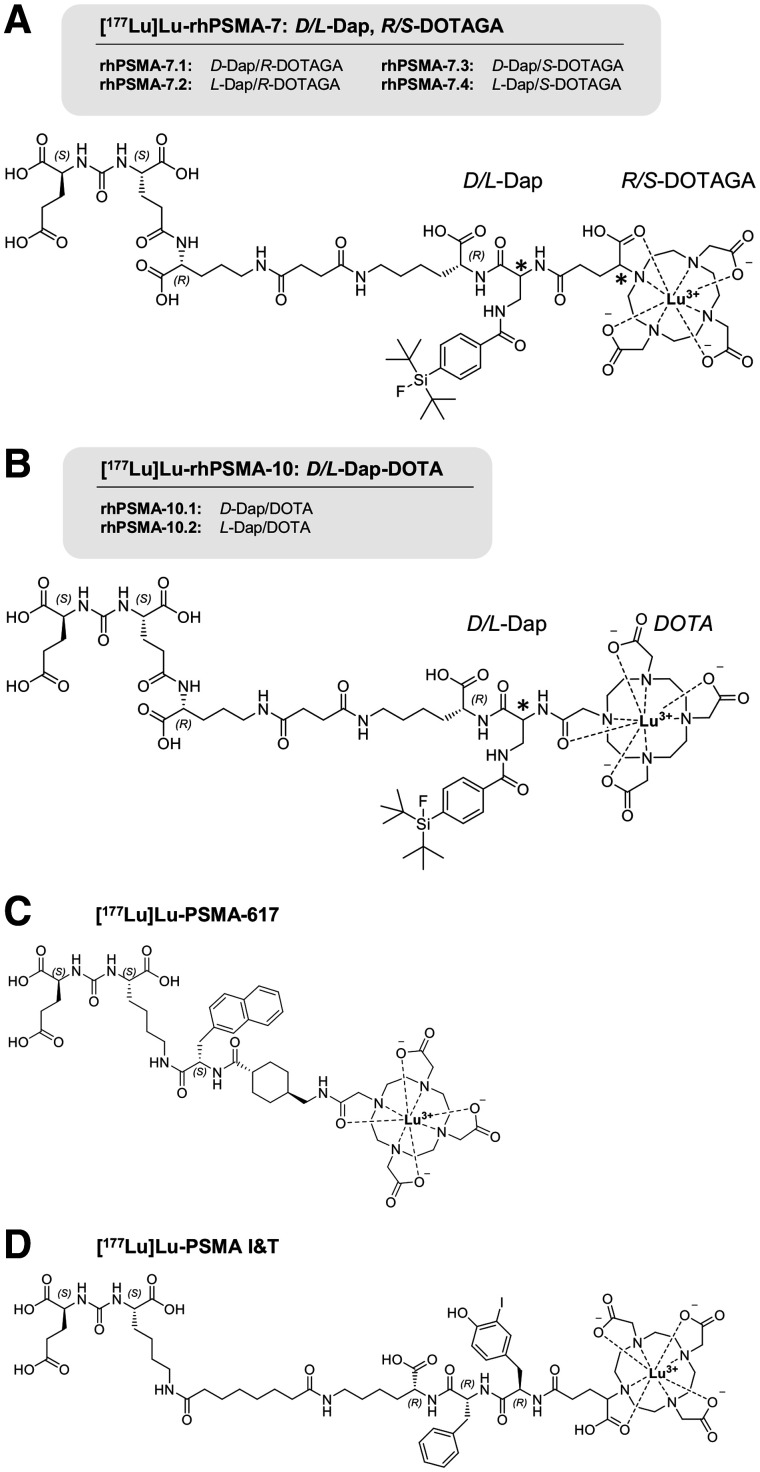
(A) rhPSMA-7 isomers differ in stereoconfiguration of diaminopropionic acid branching unit (d- or l-Dap) and glutamic acid arm at DOTAGA chelator (*R*- or *S-*DOTAGA). (B) rhPSMA-10.1 (d-Dap) and rhPSMA-10.2 (l-Dap), both equipped with DOTA chelator, also differ in stereoconfiguration of branching unit (d-or l-Dap). Well-established PSMA-addressing ligands PSMA-617 (C) and PSMA I&T (D) served as reference compounds (*1**,**2*).

## MATERIALS AND METHODS

A detailed description of the chemical synthesis of rhPSMA and the analytic instruments is provided in the supplemental materials (available at http://jnm.snmjournals.org).

### Radiolabeling

Radiolabeling with no-carrier-added ^177^Lu was performed according to the established procedures for PSMA-targeted ligands (*1**,**2*). Briefly, the precursor (1.0 nmol, 10 μL, 0.1 mM in dimethylsulfoxide) was added to 10 μL of 1.0 M aqueous NaOAc buffer (pH 5.5). Subsequently, 20–50 MBq of [^177^Lu]LuCl_3_ (specific activity > 3,000 GBq/mg at the time of radiolabeling, 740 MBq/mL, 0.04 M HCl; ITM) was added, and the mixture was filled up to 100 μL with 0.04 M HCl. The reaction mixture was heated for 20–30 min at 90°C, and the radiochemical purity was determined using radio-high-performance liquid chromatography and radio-thin-layer chromatography with 0.1 M sodium citrate buffer on instant thin-layer chromatography–silica gel chromatography paper (Agilent) and 1.0 M NH_4_OAc/dimethylformamide buffer (1/1; *v*/*v*) on thin-layer chromatography silica gel 60 F_254_ plates (Merck Millipore).

### Lipophilicity

Approximately 1 MBq of the ^177^Lu-labeled PSMA ligand was dissolved in 1 mL of a 1:1 mixture (*v*/*v*) of phosphate-buffered saline (pH 7.4) and *n*-octanol (*n* = 6). After vigorous mixing of the suspension for 3 min, the vial was centrifuged at 15,000*g* for 3 min, and 100-μL aliquots of both layers were measured in a *γ*-counter. Finally, the ratio of the radioactivity detected in the *n*-octanol sample and the phosphate-buffered saline buffer was calculated and expressed as distribution ratio log *D*_7.4_.

### Binding to HSA

Binding of ^177^Lu-labeled ligands to HSA was assessed by albumin-mediated size-exclusion chromatography (AMSEC), a novel method that has recently been developed by our group to determine the apparent molecular weight (AMW) of a compound in the presence of HSA. A dedicated and detailed description of the AMSEC method will be published elsewhere to cover the context and the development process of this method in its entirety. Briefly, a gel filtration size-exclusion column (Superdex 75 Increase 10/300 GL; fractionation range, 70–3 kDa; GE Healthcare) was calibrated as recommended by the manufacturer using a commercially available set of proteins (Gel Filtration LMW Calibration Kit; GE Healthcare). AMSEC experiments were performed by injection of the various radioligands using an HSA buffer at physiologic concentration (Biowest) as the mobile phase at room temperature. Depending on the strength of the HSA/ligand interaction during the chromatographic procedure, an injected radioligand (1.0 MBq, 10–20 GBq/μmol) can show a reduced retention time that correlates to AMWs higher than the actual, physical, molecular weight (the latter being for all investigated ligands < 2 kDa, and thus below the column fractionation range). The stronger this interaction, the longer the mean time the ligand is bound to HSA during the chromatographic process and the faster the ligand is eluted from the column. By means of calibration, the retention time can be translated into a ligand-specific AMW (expressed in kDa) as a parameter allowing quantification of the extent of HSA binding. The detection window ranges between 2.1 kDa (cutoff value, determined by [^18^F]fluoride; no HSA interaction) and 70.2 kDa (experimental molecular weight of HSA; maximum HSA interaction). [^177^Lu]Lu-rhPSMA-7.3 served as an internal standard during AMSEC studies (30.4 ± 0.5 kDa; *n* = 10).

### Affinity Determinations (Half-Maximal Inhibitory Concentration) and Internalization Studies

Competitive binding studies were determined on LNCaP cells (1.5 × 10^5^ cells in 0.25 mL/well) after incubation at 4°C for 1 h, using (((*S*)-1-carboxy-5-(4-([^125^I]iodo)benzamido)pentyl)carbamoyl)-l-glutamic acid ([^125^I]IBA)KuE; 0.2 nM/well) as the reference radioligand (*n* = 3). Internalization studies of the radiolabeled ligands (1.0 nM/well) were performed on LNCaP cells (1.25 × 10^5^ cells in 0.25 mL/well) at 37°C for 1 h and accompanied by ([^125^I]IBA)KuE (0.2 nM/well) as a reference. Data were corrected for nonspecific binding and normalized to the specific internalization observed for the reference (*n* = 3), as previously published (*8*).

### In Vivo Experiments

All animal experiments were conducted in accordance with general animal welfare regulations in Germany (German animal protection act, as amended on May 18, 2018, article 141 G, version March 29, 2017, I 626, approval 55.2-1-54-2532-71-13) and the institutional guidelines for the care and use of animals. LNCaP tumor xenografts were established in 6- to 8-wk-old male CB-17 SCID mice as described previously (*8*).

#### Biodistribution Studies

The ^177^Lu-labeled PSMA ligands (2–5 MBq; 0.1 nmol) were injected under isoflurane anesthesia into the tail vein of mice, which were euthanized 24 h after injection (*n* = 4–5). Selected organs were removed, weighed, and measured in a *γ*-counter. All rhPSMA ligands were evaluated during the same period (first quarter of 2020), whereas ^177^Lu-labeled PSMA-617 and PSMA I&T (*17*) were assessed in 2016, using the identical cell line, mouse model, and experimental procedure.

#### Small-Animal SPECT/CT Imaging

Static images of ^177^Lu-labeled ligands in euthanized mice were recorded 24 h after injection directly after blood collection, with an acquisition time of 45 min using a high-energy, general-purpose rat-and-mouse collimator and a stepwise multiplanar bed movement. For imaging studies, a VECTor4 small-animal SPECT/PET/optical imaging/CT device from MILabs was applied. Data were reconstructed using MILabs.Rec software (version 10.02) and PMOD software (version 4.0; PMOD Technologies LLC). After imaging, the mice underwent biodistribution studies.

## RESULTS

### Synthesis and Radiolabeling

Uncomplexed PSMA ligands were obtained via a solid-phase/solution-phase synthetic strategy with chemical purities of more than 97% as determined by high-performance liquid chromatography (absorbance at 220 nm). Identity was confirmed by mass spectrometry. Complexation with a 2.5-fold molar excess of LuCl_3_ led to quantitative formation of the respective lutetium-PSMA ligands, which were used for in vitro studies. ^177^Lu labeling of PSMA ligands according to standard manual procedures resulted in a radiochemical purity of more than 95%, determined by radio-high-performance liquid chromatography and radio-thin-layer chromatography (Supplemental Table 1).

### In Vitro Characterization

**FIGURE 3.**
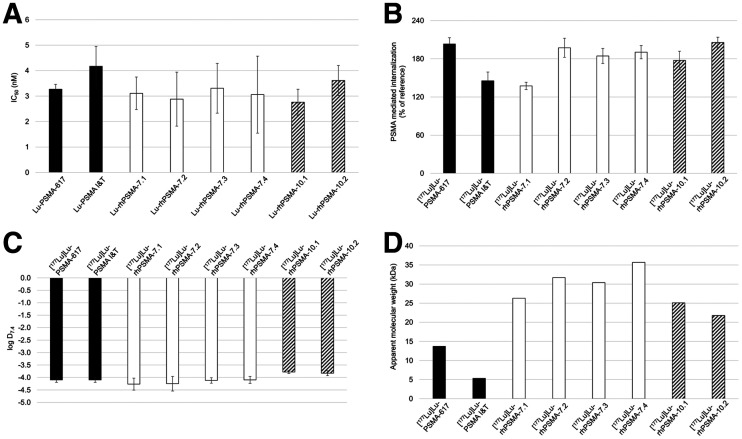
(A) Binding affinities (half-maximal inhibitory concentration [IC_50_; nM], 1 h, 4°C) of [^177^Lu]Lu-rhPSMA-7.1 to -7.4 (white; *n* = 3), [^177^Lu]Lu-rhPSMA-10.1 and -10.2 (black/white stripes; *n* = 3), and references [^177^Lu]Lu-PSMA-617 and [^177^Lu]Lu-PSMA-I&T (black; *n* = 3). (B) PSMA-mediated internalization of [^177^Lu]Lu-rhPSMA-7.1 to -7.4 (white; *n* = 3), [^177^Lu]Lu-rhPSMA-10.1 and -10.2 (black/white stripes; *n* = 3), and references [^177^Lu]Lu-PSMA-617 and [^177^Lu]Lu-PSMA I&T (black; *n* = 3) by LNCaP cells (1 h, 37°C) as percentage of reference ligand ([^125^I]IBA)KuE). (C) Lipophilicity of [^177^Lu]Lu-rhPSMA-7.1 to -7.4 (white; *n* = 6), [^177^Lu]Lu-rhPSMA-10.1 and -10.2 (black/white stripes; *n* = 6), and references [^177^Lu]Lu-PSMA-617 and [^177^Lu]Lu-PSMA I&T (black; *n* = 6), expressed as partition coefficient (log *D*_7.4_ in *n*-octanol/phosphate-buffered saline, pH 7.4). (D) AMW of [^177^Lu]Lu-rhPSMA-7.1 to -7.4 (white), [^177^Lu]Lu-rhPSMA-10.1 and -10.2 (black/white stripes), and references [^177^Lu]Lu-PSMA-617 and [^177^Lu]Lu-PSMA I&T (black), as determined by HSA-mediated size-exclusion chromatography.

Results of the in vitro evaluation of all rhPSMAs and the reference ligands PSMA-617 (*1*) and PSMA I&T (*2*) are summarized in Figure 3 and Supplemental Table 2. PSMA binding affinity (half-maximal inhibitory concentration; Fig. 3A) was high and in the low nanomolar range for all lutetium-rhPSMA ligands (range, 2.8 ± 0.5 to 3.6 ± 0.6 nM) and the 2 reference ligands ([^177^Lu]Lu-PSMA I&T, 4.2 ± 0.8 nM; [^177^Lu]Lu-PSMA-617, 3.3 ± 0.2 nM).

Slight differences between the ligands were found for the PSMA-mediated internalization into LNCaP cells (1 h, 37°C), which is expressed as a percentage of the specific internalization of the reference ligand ([^125^I]IBA)KuE (Fig. 3B). Although [^177^Lu]Lu-rhPSMA-7.1 and [^177^Lu]Lu-PSMA I&T showed the lowest internalization rates, with values of 137% ± 6% and 145% ± 14%, respectively, the other rhPSMA compounds showed an approximately 1.4-fold higher internalization (range, 177% ± 15% to 206% ± 8%), similar to that of [^177^Lu]Lu-PSMA-617 (203% ± 10%).

The ^177^Lu-labeled rhPSMA-7 isomers, as well as the references ([^177^Lu]Lu-PSMA I&T and [^177^Lu]Lu-PSMA-617), demonstrated a high and similar hydrophilicity, expressed as a partition coefficient (log *D*_7.4_; *n*-octanol and phosphate-buffered saline, pH 7.4) with values between −4.1 ± 0.1 and −4.3 ± 0.3. The DOTA conjugates [^177^Lu]Lu-rhPSMA-10.1 and -10.2 showed a slightly lower hydrophilicity, with a log *D*_7.4_ of −3.8 (Fig. 3C).

The AMW of the tracers was determined to compare the relative HSA-binding strength of the ligands. Exemplary chromatograms of AMSEC experiments showing ligand-specific retention times are provided in Supplemental Figures 1–3. Interestingly, remarkable differences were found for the AMWs of the state-of-the-art references and even among the single isomers of ^177^Lu-labeled rhPSMA-7 and rhPSMA-10 (Fig. 3D). Although [^177^Lu]Lu-PSMA I&T showed the lowest HSA interaction (AMW, 5.3 kDa), followed by [^177^Lu]Lu-PSMA-617 (AMW, 13.7 kDa), all radiohybrid ligands demonstrated an at least 1.5-fold higher AMW, with values between 21.8 and 35.7 kDa. Among the radiohybrids, the 2 DOTA conjugates, [^177^Lu]Lu-rhPSMA-10.1 and -10.2, showed the lowest AMWs (25.1 and 21.8 kDa, respectively), whereas d-Dap–configured [^177^Lu]Lu-rhPSMA-7.1 (molecular weight, 26.3 kDa) and [^177^Lu]Lu-rhPSMA-7.3 (molecular weight, 30.4 kDa) showed the lowest AMWs within the rhPSMA-7 series (AMWs of l-Dap–comprising isomers: [^177^Lu]Lu-rhPSMA-7.2, 31.7 kDa; [^177^Lu]Lu-rhPSMA-7.4, 35.7 kDa).

### In Vivo Characterization

#### Biodistribution Studies

**FIGURE 4.**
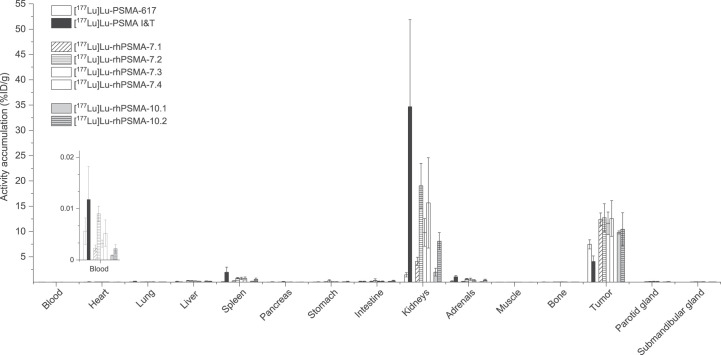
Biodistribution of [^177^Lu]Lu-rhPSMA-7.1 to -7.4, [^177^Lu]Lu-rhPSMA-10.1 and -10.2, and references [^177^Lu]Lu-PSMA-617 and [^177^Lu]Lu-PSMA I&T at 24 h after injection in male LNCaP tumor–bearing SCID mice. Data are expressed as %ID/g, mean ± SD (*n* = 4 − 5). Values of [^177^Lu]Lu-PSMA I&T were taken from previously published study by our group (*17*) that was performed under identical conditions.

Overall, the comparative biodistribution study of the ^177^Lu-labeled PSMA ligands in LNCaP tumor–bearing mice at 24 h after injection revealed a quite similar distribution pattern with high tumor uptake, fast excretion from background organs, but a varying degree of activity retention in the kidneys (Fig. 4; Supplemental Tables 3 and 4).

**FIGURE 5.**
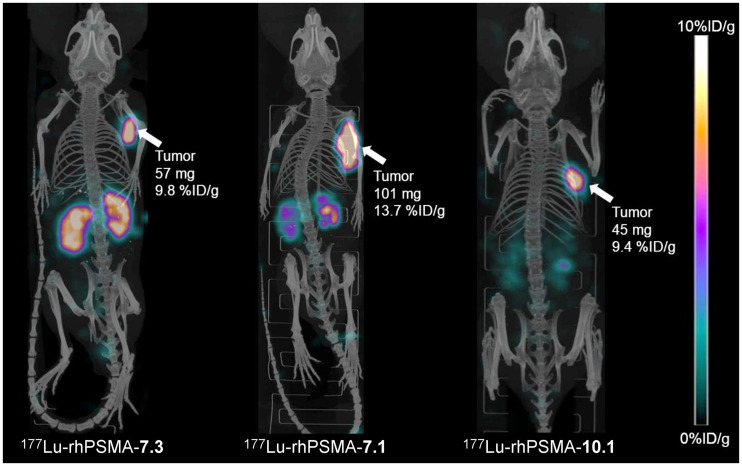
Static small-animal SPECT/CT images (maximum-intensity projections) of ^177^Lu-labeled rhPSMA-7.3, -7.1, and -10.1 in LNCaP tumor–bearing mice, euthanized 24 h after injection and imaged directly after blood collection, with acquisition time of 45 min on VECTor4 small-animal SPECT/PET/optimal imaging/CT device. Tumor weight and tracer uptake in tumor (%ID/g) were determined from subsequent biodistribution studies.

At 24 h after injection, the highest activity retention in the kidneys was found for [^177^Lu]Lu-PSMA I&T (34.7 ± 17.2 percentage injected dose [%ID]/g), whereas [^177^Lu]Lu-PSMA-617 (1.4 ± 0.4 %ID/g) and [^177^Lu]Lu-rhPSMA-10.1 (2.0 ± 0.8 %ID/g) demonstrated the fastest renal clearance. Kidney uptake of the former lead compound, [^177^Lu]Lu-rhPSMA-7.3, was found to be 9.8 ± 2.7 %ID/g, thus showing slower renal clearance than [^177^Lu]Lu-rhPSMA-7.1 (4.1 ± 0.8 %ID/g), [^177^Lu]Lu-rhPSMA-10.1 (2.0 ± 0.8 %ID/g), and [^177^Lu]Lu-rhPSMA-10.2 (8.1 ± 1.7 %ID/g). These differences are also well illustrated in the small-animal SPECT/CT images (Fig. 5). Tumor uptake was highest for all [^177^Lu]Lu-rhPSMA-7 isomers and in the range of 11.6–12.7 %ID/g, followed by [^177^Lu]Lu-rhPSMA-10.2 (10.5 ± 3.3 %ID/g) and -10.1 (9.8 ± 0.3 %ID/g), whereas the references, ^177^Lu-labeled PSMA-617 (7.5 ± 0.9 %ID/g) and PSMA I&T (4.1 ± 1.1 %ID/g), exhibited a lower tumor uptake.

#### Tumor-to-Organ Ratios

Interestingly, all radiohybrid ligands are cleared from the blood pool and background tissues with a kinetic profile that resembles that of small molecules more than that of larger proteins, despite their extensive binding to HSA. Among all radiohybrids, the highest tumor-to-blood and tumor-to-kidney ratios were found for [^177^Lu]Lu-rhPSMA-10.1 (tumor-to-blood, 11,498; tumor-to-kidney, 5.7), followed by [^177^Lu]Lu-rhPSMA-7.1 (tumor-to-blood, 5,971; tumor-to-kidney, 3.2), whereas [^177^Lu]Lu-rhPSMA-7.3 showed inferior values (tumor-to-blood, 3,843; tumor-to-kidney, 1.2). Although [^177^Lu]Lu-PSMA I&T (tumor-to-blood, 408; tumor-to-kidney, 0.2) exhibited rather slow excretion in mice, [^177^Lu]Lu-PSMA-617 showed the highest tumor-to-kidney ratio (tumor-to-blood, 1,424; tumor-to-kidney, 5.9), whereas its tumor-to-blood ratio was lower than all radiohybrid ligands (Supplemental Tables 5 and 6).

## DISCUSSION

Although it has recently been demonstrated in patients that the uptake of [^177^Lu]Lu-rhPSMA-7.3 in tumor lesions was on average 2- to 3-fold higher than that of [^177^Lu]Lu-PSMA I&T, the slower clearance resulted in a comparatively higher dose to the kidney as the organ at risk (*14*). In retrospect, this result is hardly surprising, since the selection process of the best diagnostic rhPSMA-7 isomer was based on criteria that are considered suboptimal for therapy: fast blood clearance to reach high tumor-to-background ratios at early time points, predominantly renal clearance, and high kidney retention to ensure low activity in the bladder at early time points. In contrast, the selection criteria for the best therapeutic isomer are different. Compared with today’s therapeutic ligands, a slightly delayed blood clearance is preferred. The ligand should be excreted renally while showing almost no retention in the kidneys. It must also be considered that the change in the isotope (lutetium for RLT instead of gallium for the diagnostic compound) results in a different structure and charge at the chelate–metal complex (gallium-DOTA: hexadentate, zwitterionic; lutetium-DOTA: octadentate, uncharged) (*15*). This matter obviously influences the pharmacokinetic properties of the entire ligand, as demonstrated by the prolonged clearance kinetics of the ^177^Lu-labeled “best diagnostic isomer” rhPSMA-7.3 in patients (*14*).

With the aim of addressing these therapeutic criteria and of identifying a ^177^Lu-labeled rhPSMA tracer with more favorable characteristics for RLT, we performed a coevaluation of 6 different rhPSMA ligands (4 rhPSMA-7 isomers and 2 rhPSMA-10 isomers) and compared the results with preclinical data on the 2 reference compounds, PSMA-617 and PSMA I&T.

All lutetium-complexed radiohybrid tracers and the external references, PSMA I&T and PSMA-617, demonstrated potent binding to LNCaP cells with an excellent affinity in the low-nanomolar range and high internalization rates, which did not allow prioritization of certain candidates for further evaluation.

In the context of PSMA-targeted RLT, the kidney and then the bone marrow are still considered the main organs at risk, and uptake in these should be carefully considered (*18*).

In our comparative biodistribution studies, pronounced differences in kidney uptake values were observed. Whereas our internal reference, d-Dap-*S*-DOTAGA–configured [^177^Lu]Lu-rhPSMA-7.3, showed a kidney uptake of 9.8 ± 2.7 %ID/g at 24 h after injection, the uptake of the d-Dap-DOTA derivative, [^177^Lu]Lu-rhPSMA-10.1, reached only 20% of that value (2.0 ± 0.8 %ID/g). Moreover, the stereoconfiguration of the Dap-branching unit (d-Dap or l-Dap) also resulted in a pronounced different kidney uptake, as shown for [^177^Lu]Lu-rhPSMA-7.1 (d-Dap, 4.1 ± 0.8 %ID/g) and the 5-fold higher value of the corresponding l-Dap version, [^177^Lu]Lu-rhPSMA-7.2 (19.0 ± 4.5 %ID/g). As already demonstrated in a previous study of [^18^F]Ga-rhPSMA ligands (*12*), these results impressively demonstrate once more that even small modifications in the arene binding region of PSMA-targeted ligands can have a remarkable influence on the biodistribution. In this former study, the modification of the stereoconfiguration of the Dap branching unit and the DOTAGA chelator resulted in superior pharmacokinetics of [^18^F]Ga-rhPSMA-7.3 in mice compared with the diastereomeric mixture [^18^F]Ga-rhPSMA-7. This result was confirmed in patient studies, revealing a 5-fold lower excretion of [^18^F]Ga-rhPSMA-7.3 into the bladder, a 1.6-fold lower kidney uptake, and a 1.6-fold higher tumor uptake than for the diastereomeric mixture [^18^F]Ga-rhPSMA-7 (*19*).

Even though the results of our preclinical comparison are highly promising, there are many examples in the literature that question the direct transferability of the preclinical results to clinical studies, particularly with regard to kidney clearance and kidney retention. The low kidney uptake of [^177^Lu]Lu-PSMA-617 (1.4 ± 0.4 %ID/g at 24 h after injection) in mice has often been highlighted as a major selection criterion that promoted its rapid clinical development and thus was considered a major advantage compared with [^177^Lu]Lu-PSMA I&T (kidney uptake, 34.7 ± 17.2 %ID/g at 24 h after injection) (*20*). However, in contrast to preclinical results, head-to-head comparison of both ligands in patients has impressively demonstrated a nearly identical kidney uptake and clearance kinetic of both tracers (*7*). Moreover, a similar absorbed dose to the kidney was found in dosimetry studies: 0.4 ± 0.2 to 0.8 ± 0.3 Gy/GBq for [^177^Lu]Lu-PSMA-617 (*21**–**23*) and 0.7 ± 0.2 Gy/GBq for [^177^Lu]Lu-PSMA I&T (*24*). Until further investigations improve our understanding of species-dependent renal handling of PSMA tracers, and in the absence of alternative and more valid selection criteria, the evaluation of the biodistribution in mice, including the assessment of the different excretion behavior, will remain our only viable option—although we should treat such data with appropriate caution.

Regarding important nontarget organs such as liver, muscle, and heart, all ligands demonstrated almost identical and complete clearance 24 h after injection. Even though only low activity levels were found in the blood pool for all ligands, [^177^Lu]Lu-rhPSMA-10.1 showed the best clearance of all investigated PSMA ligands, and this superior clearance is also expressed by the highest tumor-to-blood ratio (11,498): 3 times higher than for [^177^Lu]Lu-rhPSMA-7.3 and 8 times higher than for [^177^Lu]Lu-PSMA-617.

The tendency of both DOTA-conjugated [^177^Lu]Lu-rhPSMA-10 isomers to clear more quickly can at least in part be explained by the HSA-binding experiments and our newly introduced in vitro parameter, AMW. The molecular weight of a molecule is known to have a direct implication in the glomerular sieving coefficient (GSC) (as a rule of thumb, the lower the molecular weight, the higher the GSC and the faster the excretion kinetics) (*25*). Thus, the stronger the interaction of a molecule with HSA or the higher the ratio of HSA-bound ligand to free ligand, the less ligand is subjected to glomerular filtration and thus the less ligand is excreted. In our assay, this ratio is indirectly determined by calculating the AMW of each compound (details on these methods will be described elsewhere).

Looking at the AMW of ^177^Lu-labeled PSMA-617 (13.7 kDa) and PSMA-I&T (5.3 kDa) as key reference points, the 2.3-fold lower AMW of PSMA I&T appears prima facie unproportional: Kulkarni et al. could demonstrate that [^177^Lu]Lu-PSMA-617 exhibits only a marginally slower clearance in patients than does [^177^Lu]Lu-PSMA I&T (*7*). However, taking into account the nonlinear correlation of the molecular weight and GSC and the tiny changes in the GSC at low molecular weights, the differences in the AMW of PSMA I&T and PSMA-617 result in only slightly different GSCs, which explains the similar kidney excretion kinetics of these 2 ligands in patients (*25*). In contrast, the higher AMW of [^177^Lu]Lu-rhPSMA-7.3 (molecular weight, 30.4) would translate into a markedly lower GSC and thus a delayed clearance, which has been confirmed by clinical results from Feuerecker et al. (*14*). On the basis of these results, we expect that the blood clearance kinetics of [^177^Lu]Lu-rhPSMA-10.1 and -10.2 in humans will be somewhere between that of [^177^Lu]Lu-rhPSMA-7.3 and [^177^Lu]Lu-PSMA-617/PSMA-I&T. As demonstrated by Feuerecker et al. (*14*), the remarkable tumor uptake of [^177^Lu]Lu-rhPSMA-7.3 found in our preclinical experiments (*13*) could also be observed in patients (effective dose of 6.4 ± 6.7 mGy/MBq for [^177^Lu]Lu-rhPSMA-7.3 vs. 2.6 ± 2.4 mGy/MBq for [^177^Lu]Lu-PSMA I&T). Thus, we are optimistic that we will be able to obtain a similarly improved tumor uptake in patients during the clinical assessment of [^177^Lu]Lu-rhPSMA-10.1 and thus tumor doses higher than those currently obtained with the state-of-the art nonhybrid ligands (*14*).

Certainly there are also other effects determining different clearance kinetics of radiopharmaceuticals in mice and patients that must be considered—for example, species differences in drug binding to serum albumin (*26*) and differences in magnitude and binding affinities of the tracers to plasma proteins other than HSA, such as α-1-acid glycoprotein (*27*), transthyretin (*28*), or lipoproteins (*29*). Moreover, individual differences in uptake of PSMA ligands into the kidneys (*30*), varying relative proportions of hepatobiliary to renal clearance, and effects of species differences between mice and humans must be considered. In summary, however, we are optimistic that the promising biodistribution profile of [^177^Lu]Lu-rhPSMA-10.1 observed in mice, together with its low AMW, will translate into improved tumor doses and tumor-to-kidney dose ratios of this isomer in patients.

## CONCLUSION

On the basis of this preclinical comparison, [^177^Lu]Lu-rhPSMA-10.1 seems to be a promising lead for the clinical development of an rhPSMA-targeted ligand for RLT. [^177^Lu]Lu-rhPSMA-10.1 could have the potential to outperform the in vivo characteristics of the currently developed state-of-the-art PSMA targeted radioligands [^177^Lu]Lu-PSMA-617 and [^177^Lu]Lu-PSMA-I&T in men. Clinical studies are required to demonstrate whether our newly introduced additional preclinical selection criterion, the AMW, could be a valuable parameter for the future development of further therapeutic radiopharmaceuticals with optimally adjusted clearance kinetics.

## DISCLOSURE

Hans-Jürgen Wester, Alexander Wurzer, Jan-Philip Kunert, Sebastian Fischer, and Veronika Felber are listed as inventors in patent applications for some types of therapeutic rhPSMA. Hans-Jürgen Wester receives funding from the SFB 824 (Deutsche Forschungsgemeinschaft, Bonn, Germany, Sonderforschungsbereich 824, project B11 and Z). Hans-Jürgen Wester is a founder and shareholder of, and a scientific advisor for, Scintomics GmbH, Fuerstenfeldbruck, Germany. Wolfgang Weber is a consultant for Blue Earth Diagnostics Ltd. No other potential conflict of interest relevant to this article was reported.

## Notes

KEY POINTS **QUESTION:** Which ^177^Lu-labeled rhPSMA ligand shows the best characteristics for RLT of prostate cancer? **PERTINENT FINDINGS:** In preclinical experiments, [^177^Lu]Lu-rhPSMA-10.1 demonstrated fast clearance kinetics from healthy tissues while preserving high tumor uptake. **IMPLICATIONS FOR PATIENT CARE:** Preclinical data indicate more favorable pharmacokinetics for [^177^Lu]Lu-rhPSMA-10.1 than for [^177^Lu]Lu-PSMA-617 and [^177^Lu]Lu-PSMA-I&T for RLT, a finding that has to be investigated in prospective clinical studies.
